# Supplementary material for: Tetraethylenepentamine-Coated β Cyclodextrin Nanoparticles for Dual DNA and siRNA Delivery
Source: Pharmaceutics. 2022 Apr 23;14(5):921. doi: 10.3390/pharmaceutics14050921 (PMC9145619; doi:10.3390/pharmaceutics14050921)
Supplement: Supplementary file 1 [file pharmaceutics-14-00921-s001.zip › pharmaceutics-1644762-supplementary.pdf]

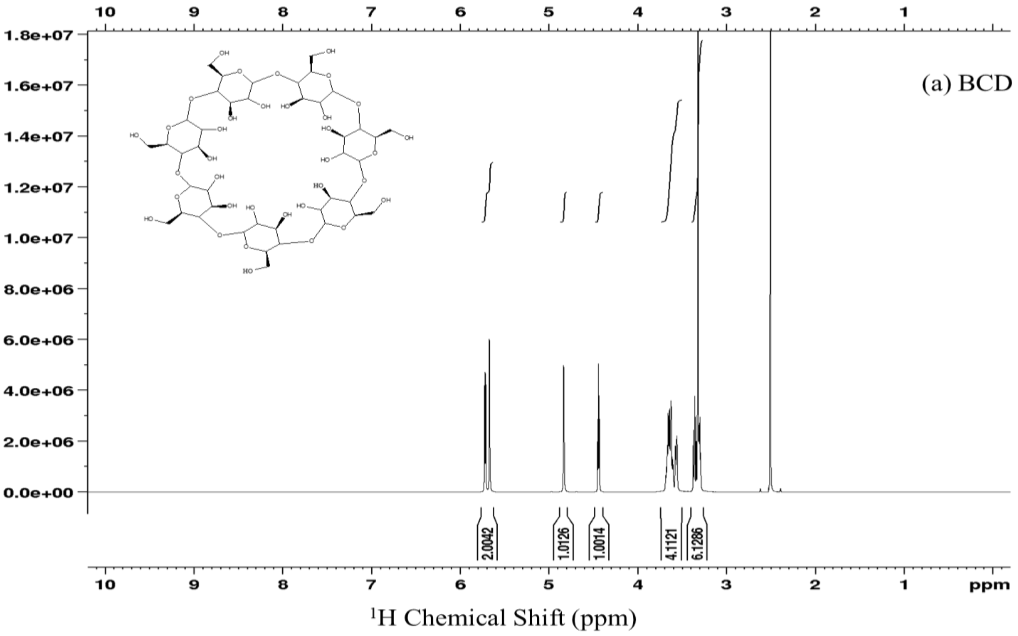
(a) BCD
2.0042
1.0126
1.0014
4.1121
6.1286
ppm
¹H Chemical Shift (ppm)

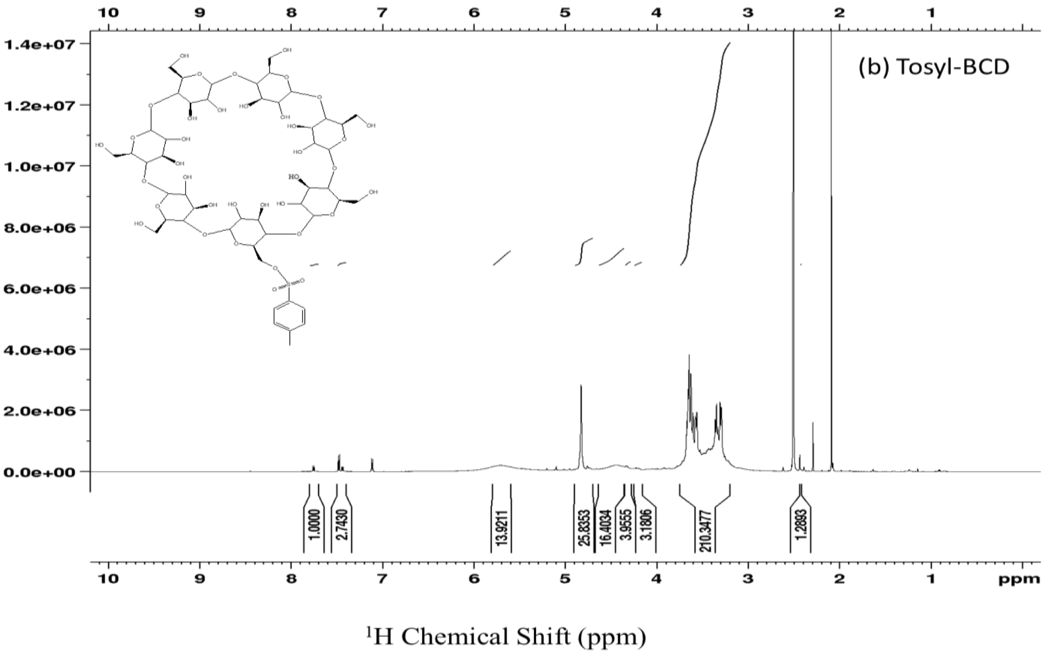
(b) Tosyl-BCD
1.0000
2.7430
13.9211
25.8353
16.4034
3.9555
3.1806
210.3477
1.2883
ppm
¹H Chemical Shift (ppm)

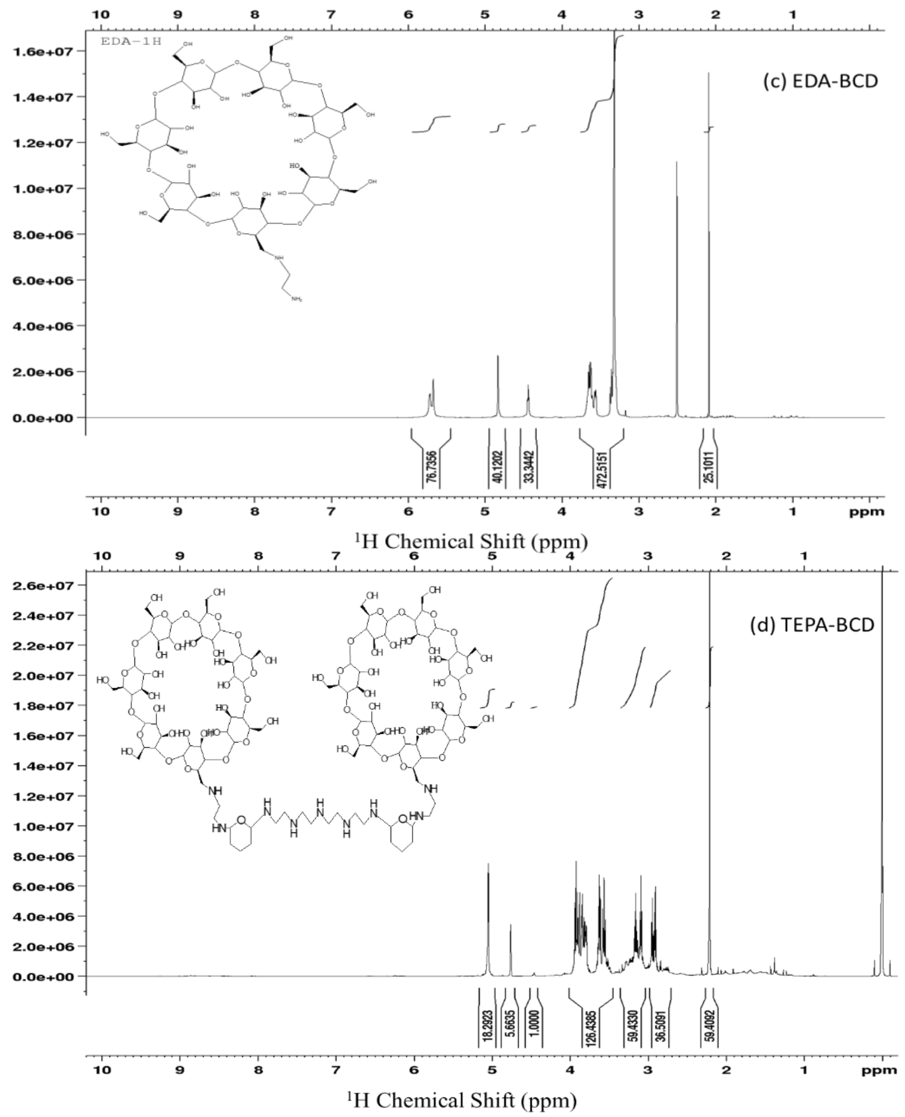

Figure S1. ^{1}H spectra of (**a**) BCD, (**b**) Tosyl-BCD, (**c**) EDA-BCD, (**d**) TEPA-BCD.

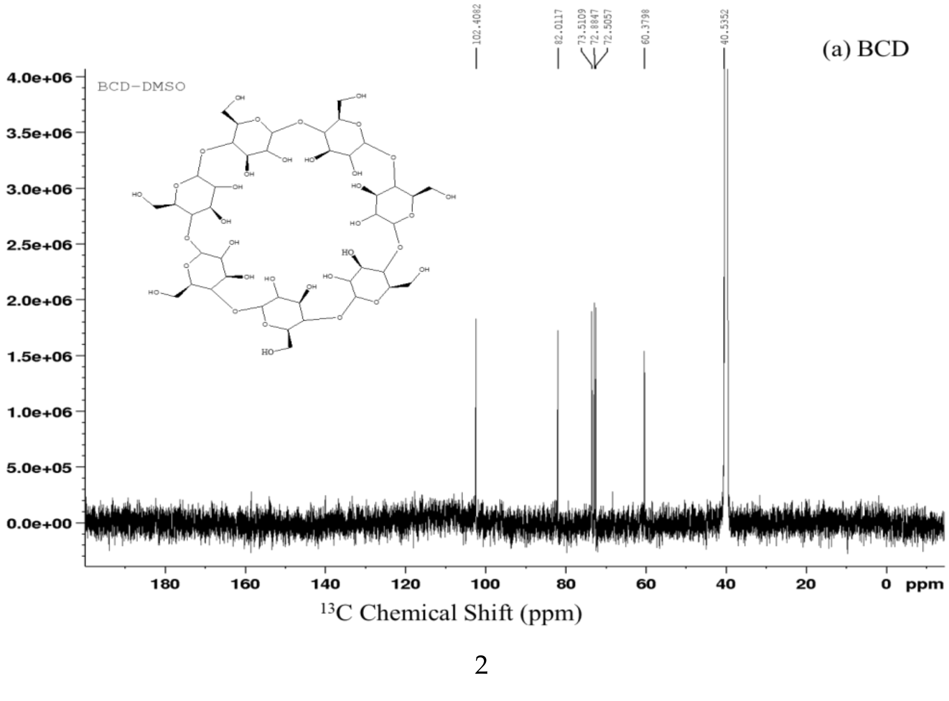

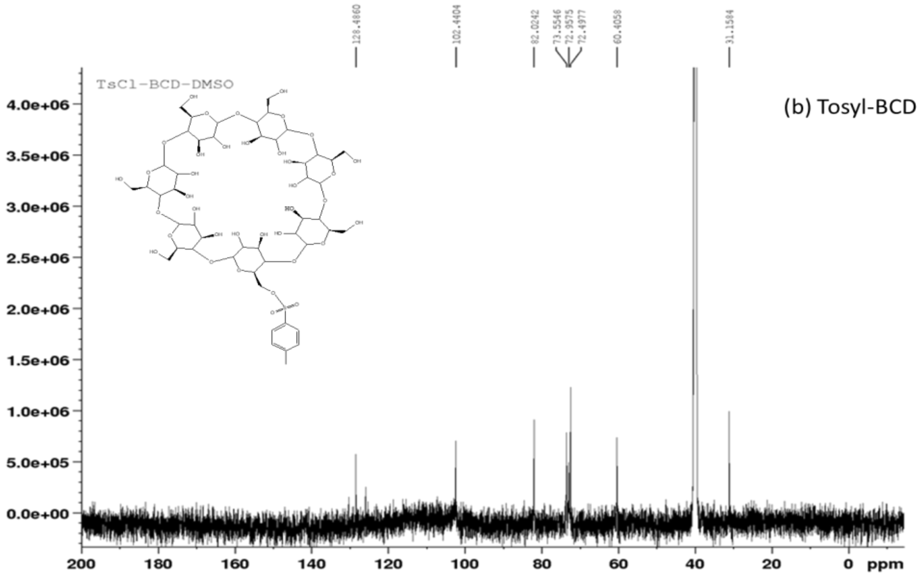

^{13}C Chemical Shift (ppm)

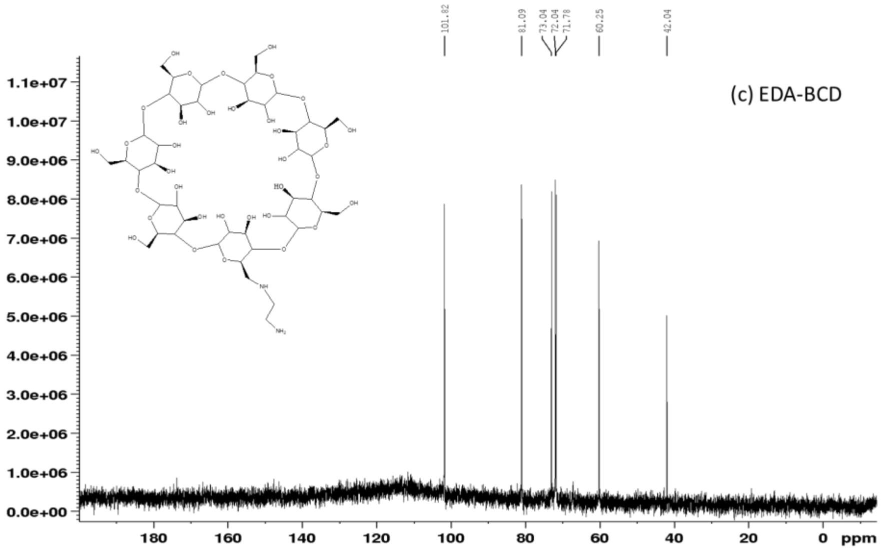

^{13}C Chemical Shift (ppm)

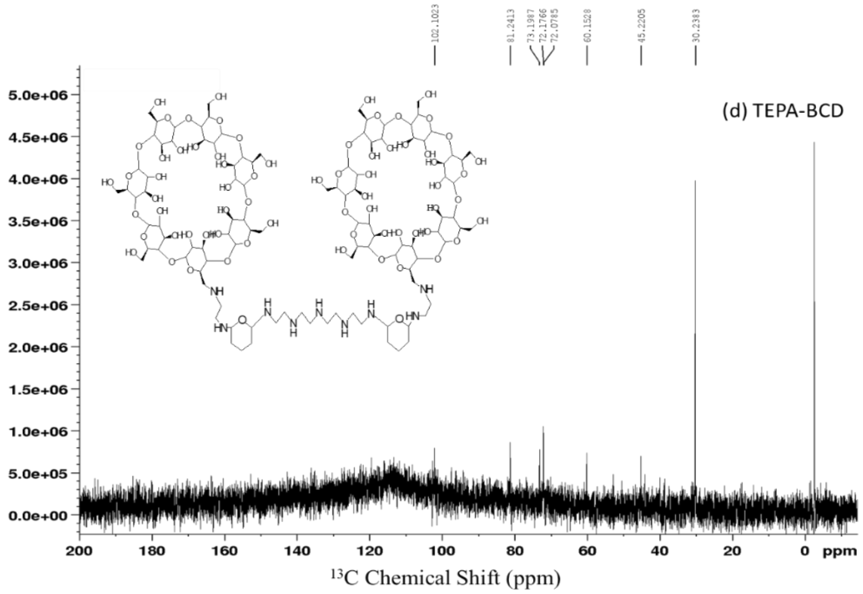

Figure S2. ^{13}C spectra of (**a**) BCD, (**b**) Tosyl-BCD, (**c**) EDA-BCD, (**d**) TEPA-BCD.

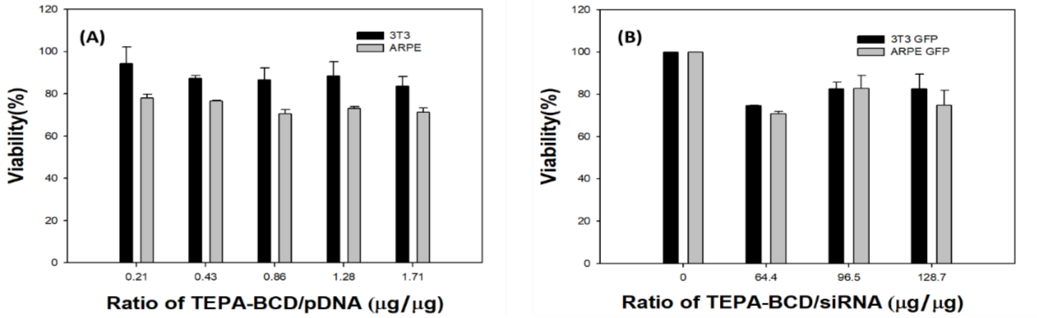

Figure S3. Cell viability of 3T3 and ARPE (**A**), and their GFP-expressing cells (**B**) after 72 h treatment of TEPA-BCD complexes.

ARPE cells	0 hr	4 hr	8 hr	24hr	48hr
Control	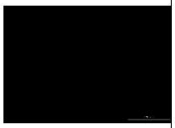	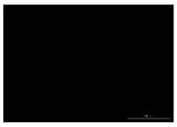	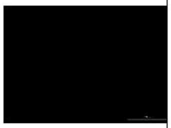	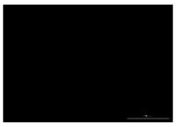	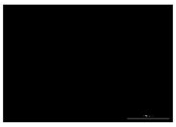
TEPA-BCD/ plasmid DNA	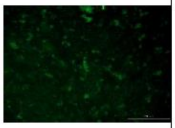	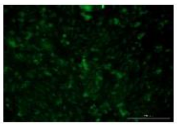	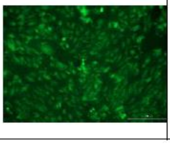	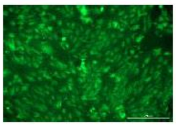	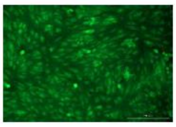

Figure S4. Time-course fluorescence imaging demonstrated GFP upregulation in ARPE cells after TEPA-BCD/plasmid DNA transfection. Control groups were nontreated cells.

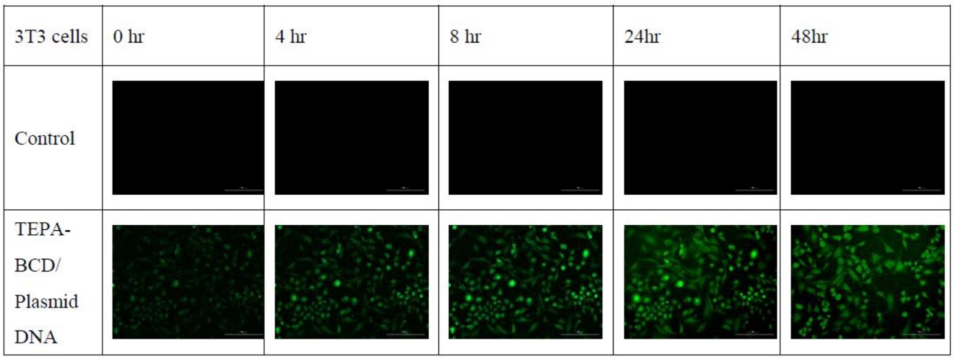

Figure S5. Time-course fluorescence imaging demonstrated GFP upregulation in 3T3 cells after TEPA-BCD/plasmid DNA transfection. Control groups were nontreated cells.

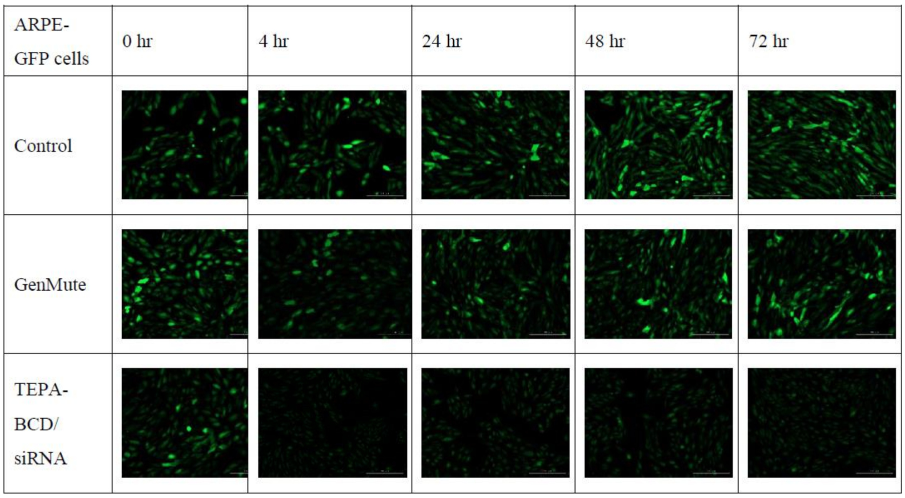

Figure S6. Time-course fluorescence imaging demonstrated GFP silencing in ARPE-GFP cells after TEPA-BCD/siRNA transfection. Control groups were nontreated cells. GenMute served as a positive control.

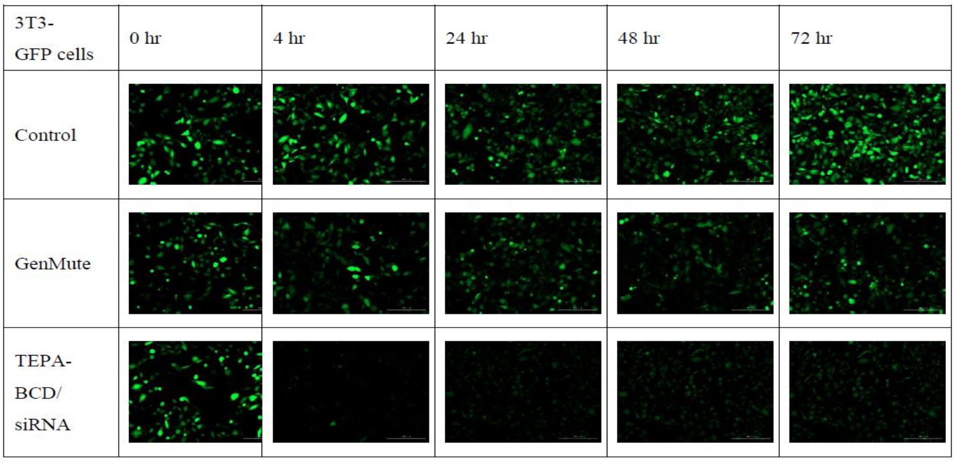

Figure S7. Time-course fluorescence imaging demonstrated GFP silencing in 3T3-GFP cells after TEPA-BCD/siRNA transfection. Control groups were nontreated cells. GenMute served as a positive control.
